# Pulling the lever in a hurry: the influence of impulsivity and sensitivity to reward on moral decision-making under time pressure

**DOI:** 10.1186/s40359-024-01773-y

**Published:** 2024-05-14

**Authors:** Fiorella Del Popolo Cristaldi, Grazia Pia Palmiotti, Nicola Cellini, Michela Sarlo

**Affiliations:** 1 Department of General Psychology, University of Padua, Via Venezia 8, Padua, 35131 Italy; 2 WFI - Ingolstadt School of Management, Catholic University of Eichstätt-Ingolstadt, Auf d. Schanz 49, 85049 Ingolstadt, Germany; 3 Padua Neuroscience Center (PNC), University of Padua, Via Orus 2/B, Padua, 35129 Italy; 4 Department of Communication Sciences, Humanities and International Studies, University of Urbino Carlo Bo, Via Aurelio Saffi 2, Urbino, 61029 Italy

**Keywords:** Moral dilemmas, Time pressure, Impulsivity, Decision-making, BIS-BAS

## Abstract

**Background:**

Making timely moral decisions can save a life. However, literature on how moral decisions are made under time pressure reports conflicting results. Moreover, it is unclear whether and how moral choices under time pressure may be influenced by personality traits like impulsivity and sensitivity to reward and punishment.

**Methods:**

To address these gaps, in this study we employed a moral dilemma task, manipulating decision time between participants: one group (*N* = 25) was subjected to time pressure (TP), with 8 s maximum time for response (including the reading time), the other (*N* = 28) was left free to take all the time to respond (noTP). We measured type of choice (utilitarian vs. non-utilitarian), decision times, self-reported unpleasantness and arousal during decision-making, and participants’ impulsivity and BIS-BAS sensitivity.

**Results:**

We found no group effect on the type of choice, suggesting that time pressure per se did not influence moral decisions. However, impulsivity affected the impact of time pressure, in that individuals with higher cognitive instability showed slower response times under no time constraint. In addition, higher sensitivity to reward predicted a higher proportion of utilitarian choices regardless of the time available for decision.

**Conclusions:**

Results are discussed within the dual-process theory of moral judgement, revealing that the impact of time pressure on moral decision-making might be more complex and multifaceted than expected, potentially interacting with a specific facet of attentional impulsivity.

**Supplementary Information:**

The online version contains supplementary material available at 10.1186/s40359-024-01773-y.

## Background

Making timely moral decisions is a real challenge, as emerged during the COVID-19 pandemic where physicians and nurses were forced to quickly choose which patients to treat first under limited healthcare resources.

Sacrificial moral dilemmas are reliable experimental probes to study the contribution of cognitive and emotional processes to moral decision-making [1]. In these studies, participants are confronted with life-and-death hypothetical scenarios where they have to decide whether to endorse or reject the utilitarian choice of killing one person to save more lives. In the classic Trolley dilemma, the utilitarian option requires pulling a lever to redirect a runaway trolley, which would kill five workmen, onto a sidetrack where it will kill only one person; in the Footbridge version, it requires pushing one large man off an overpass onto the tracks to stop the runaway trolley. Research consistently showed that most people respectively endorse and reject the utilitarian resolution in trolley- and footbridge-like dilemmas, despite the identical cost-benefit trade-off [1–3].

According to the dual-process model of moral judgement [1], responses to moral dilemmas are driven by the outcomes of a competition between cognitive and emotional processes. In the Footbridge case, a strong emotional aversive reaction to causing harm to one person overrides a cognitive-based analysis of saving more lives, driving toward the rejection of the utilitarian resolution because harming someone is perceived as an intended means to an end. Instead, in the Trolley case, a lower emotional engagement allows the deliberate cost-benefit reasoning to prevail and drive toward the utilitarian choice since harming someone is perceived as an unintended side effect. Therefore, dilemma resolutions vary depending on how much each dilemma type elicits aversive emotions, so that the more emotional processes are engaged the higher the likelihood of rejecting utilitarian choices. Unsurprisingly, in scenarios where the decision-maker’s own life is at stake (“personal involvement”), this pattern reverses, so that a strong negative emotional reaction to self-sacrifice pushes towards utilitarian, self-protective behaviour [4].

Time is a key feature of high-stakes human choices. Time pressure alters decision-making by increasing reliance on emotional states [5]. Previous research in moral decision-making has demonstrated that time pressure affects the outcomes and the processes involved in moral judgement, as it is assumed to reduce the time for the cost-benefit calculation letting emotional processes prevail. This led to a reduced proportion of utilitarian choices [6–10], and a decreased willingness to self-sacrifice in dilemmas with personal involvement [11]. However, evidence remains mixed, with some studies suggesting that reduced decision times are associated with a higher proportion of utilitarian choices [12, 13], and other studies finding null results [14]. Moreover, very few studies have investigated if these phenomena are influenced by personality traits known to affect how people make decisions. Among these, impulsivity and motivational drives towards action/inhibition seem particularly relevant.

Impulsivity involves multiple cognitive and behavioural domains (e.g., inability to reflect on choices’ outcomes, to defer rewards, and to inhibit prepotent responses; [15]) that are strongly involved in decision-making. Beyond research on psychopathy, studies investigating the role of impulsivity in moral dilemmas are surprisingly scarce. Within moral judgments, higher impulsivity should reduce the engagement of deliberative processes, thereby allowing emotional processes to prevail. Nonetheless, previous studies measuring impulsivity in moral judgement tasks have found no effects of impulsivity on the type of resolutions taken [16–18], and to our knowledge, no study has manipulated decision times.

Motivational drives towards action/inhibition, namely the Behavioural Inhibition and Activation Systems (BIS/BAS), are worthy of investigation, too. Indeed, the BIS is sensitive to signals of punishment, inhibiting behaviours leading to negative outcomes or potential harm; whereas the BAS is sensitive to reward, driving to behaviours resulting in positive outcomes [19]. Within moral dilemmas, “reward” corresponds to the maximisation of lives saved, thereby driving towards utilitarian resolutions. Consistently, previous research [20] showed that higher BAS individuals tended to make an overall higher number of utilitarian choices, while higher BIS participants tended to reject utilitarian resolutions, particularly in footbridge-like dilemmas. Notably, without time constraints no effects of BIS-BAS emerged on response times.

In summary, there is strong evidence that cognitive-emotional conflict drives moral decisions in sacrificial dilemmas and that reducing decision time can further affect moral choices. However, the direction of this effect is still unclear, as well as if impulsivity and BIS-BAS sensitivity might influence these processes. To address these gaps, in our study, we used a standardised set of moral scenarios to investigate the effect of impulsivity and BIS-BAS sensitivity on moral decision-making under time pressure. We manipulated decision time between participants, as it has been successfully done by the majority of studies manipulating time pressure in a moral dilemma task (e.g [6–9]). A within-subjects design, conversely, may not have been appropriate because it could have generated a sequential effect in the responses (cf [21, 22]): the speeding effect of the time pressure condition could have extended to the condition with no time pressure, potentially undermining the effectiveness of the manipulation. Moreover, we measured impulsivity and BIS/BAS sensitivity as well as self-reported valence and arousal experienced during decision-making. Consistently with the dual-process model, in the time pressure group, we expected to find faster response times, higher arousal and unpleasantness ratings, and lower proportions of utilitarian choices [6–10]. As for the effect of impulsivity and BIS-BAS sensitivity, the literature is less conclusive in guiding stringent confirmatory hypotheses. Within the dual-process framework, we might hypothesise that individuals with higher impulsivity would exhibit a greater tendency towards emotionally-driven responses, particularly under time pressure. Time constraints might hinder a careful evaluation of different options and decision outcomes by increasing emotional activation or depleting the cognitive resources available for decision-making. This might lead to a lower endorsement of utilitarian choices and/or to an increase in self-protective behaviours in dilemmas involving personal involvement. In line with [20] Moore et al. (2011), individuals with higher BAS sensitivity might show an overall propensity towards utilitarian resolutions, while BIS-reactive individuals might show the opposite, and this trend should be reversed in dilemmas with personal involvement.

## Methods

### Participants

Sixty healthy university students (37 F) were recruited to voluntarily participate in the study. They had no history of psychiatric or neurological disorders, nor prior knowledge of moral dilemmas. The sample size was based on previous studies manipulating time pressure in moral dilemma tasks [6, 8], and allowed to reach a 96% post-hoc power (α = 0.05, f = 0.50).

Participants were randomly assigned to either the time pressure (TP, *N* = 30) or no time pressure (noTP, *N* = 30) group. Data from 6 participants were discarded because of deviations from instructions during data collection (e.g., reversed response scales, not keeping the fingers on the computer keys during the task). Data from 1 participant was discarded according to the a-priori criterion of missing responses in more than 20% of the trials. The final sample included 53 participants (TP group = 25, F = 15, age M = 22 years, SD = 1.55 years, range = 20–25; noTP group = 28, F = 17, age M = 21.9 years, SD = 1.77 years, range = 19–25).

All participants gave written consent before participation. The study was submitted and approved by the Ethical Committee for the Psychological Research of the University of Padua (protocol n. 2105) and conducted in accordance with the Declaration of Helsinki.

### Stimulus material

A set of 75 moral dilemmas [4] was administered to each participant. This consisted of 60 experimental dilemmas and 15 filler dilemmas. Experimental dilemmas included 30 trolley- and 30 footbridge-like dilemmas, of which 15 with personal involvement and 15 without personal involvement. Filler dilemmas were similar to experimental dilemmas but described non-deathly moral issues (e.g., stealing, lying, being dishonest), and were included to avoid automaticity in responding due to habituation to deathly scenarios. This condition was not analysed and will not be discussed further here.

Dilemmas were presented randomly within 3 blocks of 25 trials each (10 footbridge-like, 10 trolley-like, and 5 filler dilemmas). Each dilemma was presented as text, in white type against a grey background, through a series of two screens. The first described the scenario, in which some threat is going to cause the death of a group of people; the second described the hypothetical action (utilitarian option, namely saving more lives), in which the agent kills one individual to save the group of people. Participants had to choose whether or not enacting this behaviour by pressing the corresponding key on the computer keyboard.

Stimuli were presented on a 19-inch computer screen at 100 cm distance. Stimuli were presented with E-prime software [23].

### Procedure

Upon arrival, participants were given information about the experiment, and they signed the informed consent. Then, they were asked to fill out the State-Trait Anxiety Inventory (STAI Form Y-2) [24] and the Beck Depression Inventory (BDI-II) [25]. Since anxiety and depression interact with emotional reactivity and with decision-making under time pressure [26], we decided to measure (and control for them) in our experiment.

Afterwards, participants sat in a sound-attenuated room where instructions for the task were given. Specifically, they were asked to identify with the main character of the scenarios. Each trial began with the scenario, that participants could read at their own pace. After pressing the spacebar, the utilitarian option was presented for a maximum of 8 s in the TP group and for an unlimited time in the noTP group. Participants were asked to read the proposed action and decide whether to choose it or not by pressing one of two computer keys marked as “YES” or “NO”.

In the TP group participants had a limited time to respond, as indicated by a white bar located on the upper side of the screen above the text, decreasing in size every second and disappearing when time ran out. Instructions stressed to respond within the limited time indicated by the bar. If participants failed to respond within the allotted time the next scenario would appear. In the noTP group participants were instructed to respond when they reached a decision, having as much time as they wanted to decide. In both groups, response times were recorded from the onset of the utilitarian option on the screen.

After their response, participants were required to rate how they felt while they were deciding using a computerised version of the Self-Assessment Manikin (SAM) [27], displaying the 9-point scales of valence (unpleasantness/pleasantness) and arousal (calm/activation), with higher scores indicating higher pleasantness and higher arousal. Then, the next scenario was presented. After each block of trials, participants could take a break to avoid fatigue. Before starting the experimental session, each participant familiarised with the task through two practice trials to check that they understood the instructions properly. After the experimental session, participants were asked to fill out the Barratt Impulsiveness Scale (BIS-11) [28] and the BIS-BAS Scales [29].

The BIS-11 is a 30-item self-report questionnaire measuring impulsivity. It is rated on a 4-point Likert scale from 1 = rarely/never to 4 = almost always/always. The total scores range from 30 to 120, with higher total scores reflecting higher levels of impulsivity. The BIS-11 comprises six first-order subscales of attention (e.g., “focusing on the task at hand”), motor impulsiveness (e.g., “acting on the spur of the moment”), self-control (e.g., “planning and thinking carefully”), cognitive complexity (e.g., “enjoy challenging mental tasks”), perseverance (e.g., “a consistent lifestyle”), and cognitive instability (e.g., “thought insertions and racing thoughts”). These fell under three second-order subscales: attentional impulsiveness (attention and cognitive instability), motor impulsiveness (motor impulsiveness and perseverance), and non-planning impulsiveness (self-control and cognitive complexity).

The BIS-BAS scales are a self-report measure of BIS-BAS sensitivity containing a 7-item BIS subscale and a 13-item BAS factor comprising 3 subscales. It is rated on a 5-point Likert scale from 1 = “does not describe me at all” to 5 = “describes me completely”, with higher scores indicating higher BIS-BAS sensitivity. The BIS subscale includes items regarding reactions to the anticipation of punishment. The BAS factor assesses how people respond to potentially rewarding events and comprises three subscales: Reward Responsiveness (5 items regarding the positive responses to anticipated or actual reward), Drive (4 items pertaining to pursuing desired goals), and Fun Seeking (4 items referring to desiring new rewards and willing to approach a current potentially rewarding event).

### Data analysis

The study has a 2 (*group*, between-subjects: TP vs. noTP) x 2 (*dilemma type*, within-subjects: trolley-like vs. footbridge-like) x 2 (personal *involvement*, within-subjects: no involvement vs. involvement) mixed design. We measured as dependent variables (DVs): *type of choice* (utilitarian vs. non-utilitarian), choice *response times* (in msec), *valence*, and *arousal* ratings. We chose not to include the type of choice (utilitarian vs. non-utilitarian) as an additional fixed factor in our analysis, although we acknowledge that it may be a factor of interest, because in our sample the number of trials where participants opted for utilitarian resolutions was not comparable to the number of trials where participants rejected utilitarian resolutions within each dilemma type. Thus, a statistical comparison between the two types of choice would have been unreliable. However, for the sake of completeness, we provide in the Supplementary Material descriptive statistics (Table S1) and plots (Figure S1) regarding choice response times, valence and arousal ratings as a function of group (TP vs. noTP), dilemma type (trolley- vs. footbridge-like) and type of choice (utilitarian vs. non-utilitarian).

Data were pre-processed according to the following a-priori criteria: trials with missing values and with response times ≤ 150 msec were discarded (∼ 23%), response times were log-transformed to account for their skewed distribution [30], and questionnaire scores were mean-centred.

Analyses were performed using R software. Outliers were detected through median absolute deviation values (MAD > 3) computed on choice, choice response times, valence, and arousal ratings. We identified 6 univariate outliers. However, visual inspection of their ratings showed that they were characterised only by slightly different values than other participants. Since none of them significantly impacted the models’ estimates (as assessed through Cook’s distance, see below), we decided to keep them in data analysis. Data from 53 participants entered data analysis.

For each DV we fitted a (Generalised) Linear Mixed-effects Model ((G)LMM) with individual random intercept, *group*, *dilemma type*, personal *involvement*, and their interaction as fixed factors. We used a binomial family for the GLMM on choice and a Gaussian family for the LMMs on the remaining DVs. BIS-11 and BIS-BAS scores were added as covariates in separate models, controlling for STAI and BDI-II scores in each model. When a significant effect of a questionnaire predictor was found, additional models testing the slopes of questionnaire trends for each level of the fixed factors (group and dilemma type) were performed.

Influential cases (*N* = 0) were evaluated through Cook’s distance (> 1). GLMMs effects were tested through Type II Analysis of Deviance, while LMMs effects were tested by means of *F*-test and *p*-values calculated via Satterthwaite’s degrees of freedom method (α = 0.05). All post-hoc pairwise comparisons were tested through estimated marginal means or trends contrasts, adjusted for multiple comparisons with the False Discovery Rate (FDR) method. For each model, in the Supplementary Material Tables S2-S5 we report the estimated parameters with 95% CI, marginal, and conditional R^2^.

## Results

### Descriptive statistics

Descriptive statistics are summarised in Table 1.

**Table 1:** Mean (M) and standard deviation (SD) of the main research variables

Group	Dilemma type	Utilitarian choices (proportion, M ± SD)	Choice RTs (msec, M ± SD)	Valence (1–9, M ± SD)	Arousal (1–9, M ± SD)
TP (*N = 25)*	Trolley-like	0.71 ± 0.45	5731 ± 1102	2.9 ± 2	6.5 ± 2.3
	Footbridge-like	0.22 ± 0.42	4941 ± 1309	3 ± 2	6.3 ± 2.3
noTP (*N = 28)*	Trolley-like	0.68 ± 0.47	10,354 ± 6250	2.6 ± 1.5	6.4 ± 2.0
	Footbridge-like	0.22 ± 0.41	8849 ± 6305	2.8 ± 1.6	6.3 ± 2.1

Notes. TP: Time Pressure group; noTP: no Time Pressure group; RT: Response Time

### Proportion of utilitarian choices

**Fig. 1:**
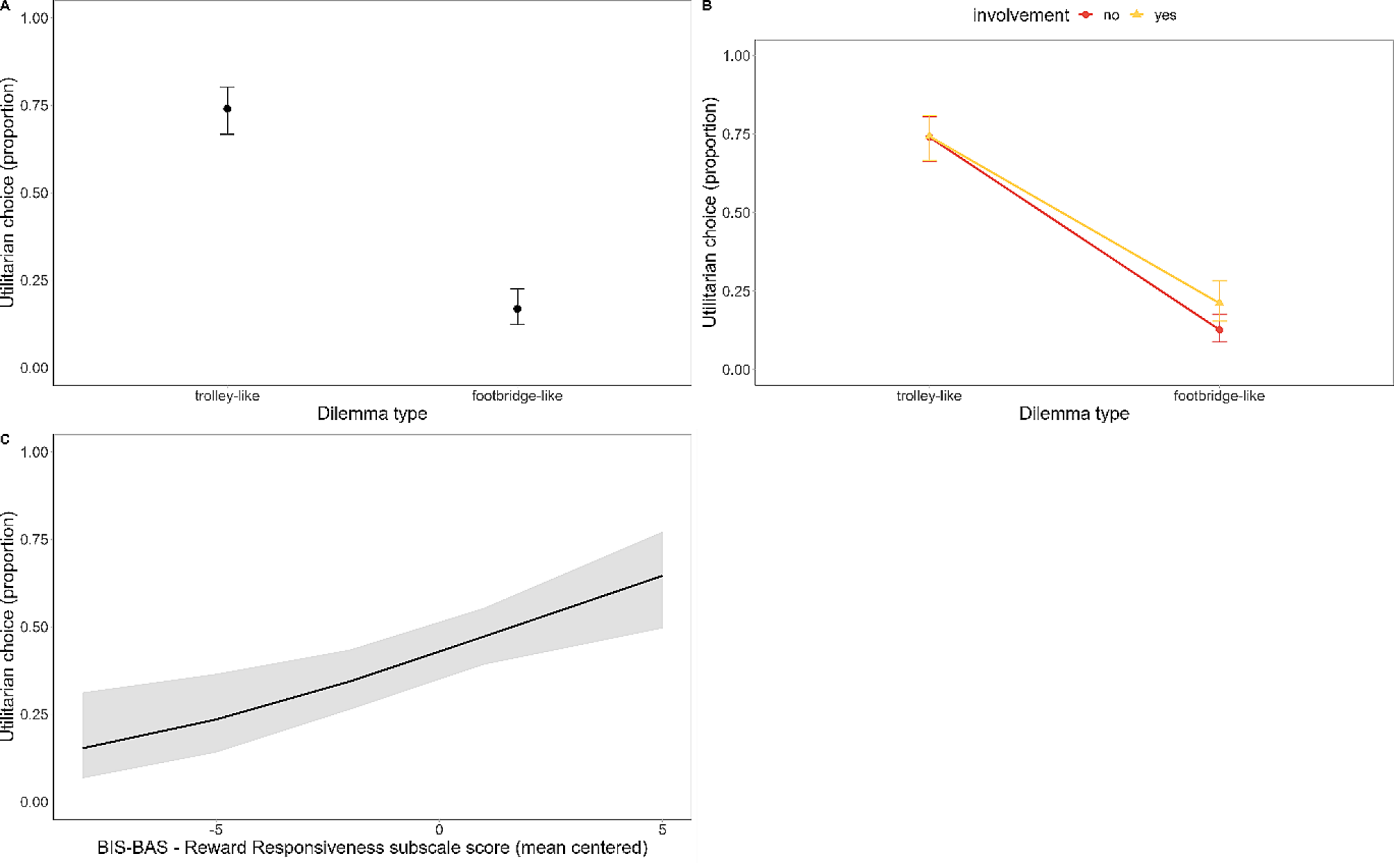
(**A**) Utilitarian choices as a function of the Dilemma type. (**B**) Utilitarian choices as a function of Personal Involvement. (**C**) Relation between utilitarian choices and BIS-BAS Reward Responsiveness subscale scores in the whole sample. Error bars (and grey area) represent standard errors of the means

The model on choices (R^2^ marginal = 0.277; R^2^ conditional = 0.506; Fig. 1A; Supplementary Material Table S2) did not show significant group effects (χ^2^ (1) = 1.05, *p* = .777). A main effect of *dilemma type* (χ^2^ (1) = 647.064, *p* < .001) was observed, with trolley-like dilemmas eliciting a higher proportion of utilitarian choices than footbridge-like dilemmas (trolley vs. footbridge: 2.68, SE = 0.104, *z* = 25.63, *p* < .001). We also found a main effect of *involvement* (χ^2^ (1) = 9.36, *p* = .002), better specified by a significant *dilemma type* × *involvement* interaction (χ^2^ (1) = 10.13, *p* = .002; Fig. 1B). Dilemmas with personal involvement elicited a higher proportion of utilitarian choices than dilemmas without personal involvement only in footbridge-like dilemmas (trolley no involvement vs. involvement: -0.026, SE = 0.126, *z* = -0.207, *p* = .836; footbridge no involvement vs. involvement: -0.610, SE = 0.138, *z* = -4.424, *p* < .001). Lastly, we found significant effects of BIS-BAS scores: regardless of *group* and *dilemma type*, and controlling for STAI and BDI-II scores, higher Reward Responsiveness subscale scores predicted a higher proportion of utilitarian choices (R^2^ marginal = 0.310; R^2^ conditional = 0.497; χ^2^ (1) = 9.35, *p* = .002; β = 0.181, SE = 0.06, *z* = 3.27, *p* = .001; Fig. 1C).

### Choice response times

**Fig. 2:**
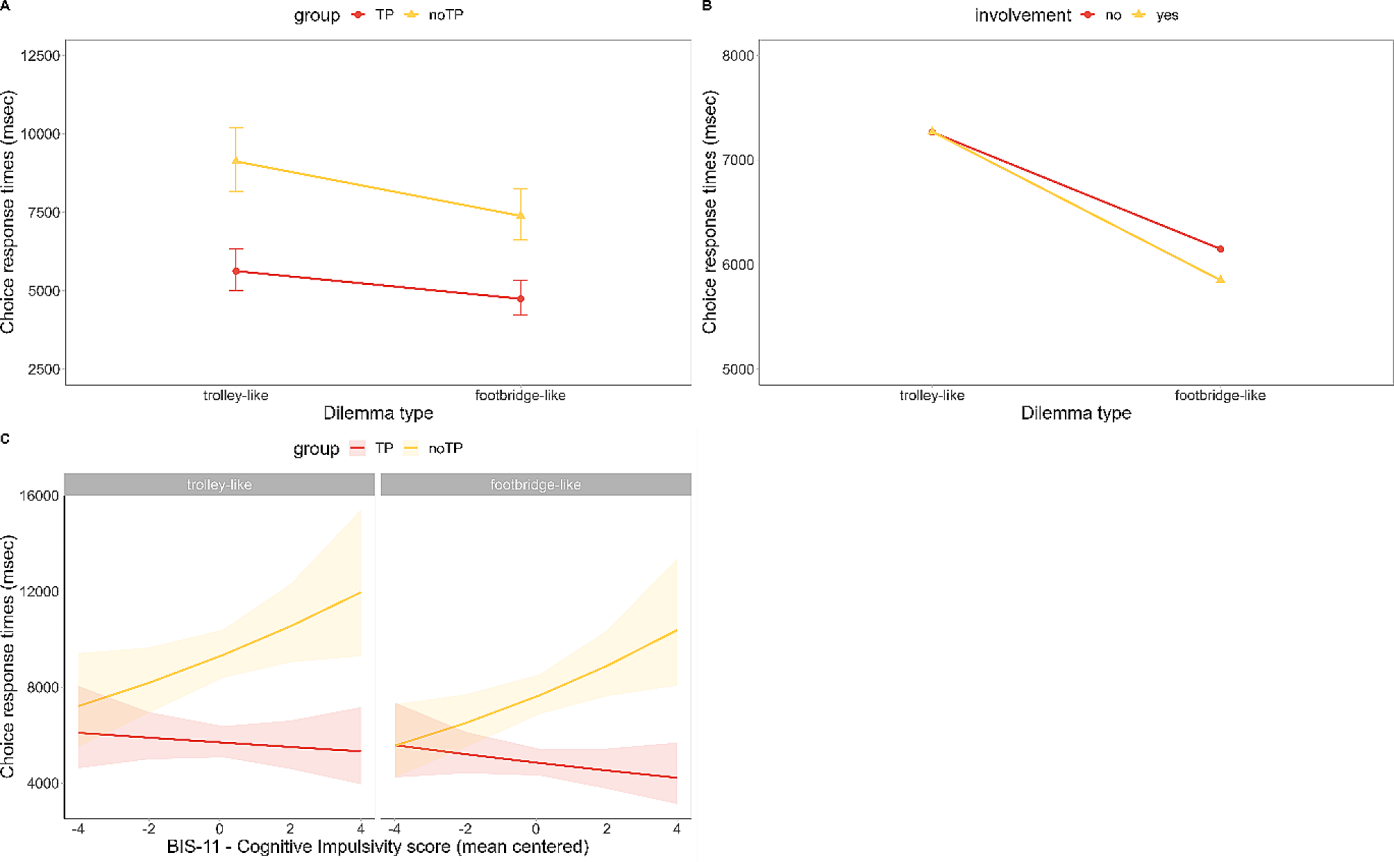
(**A**) Choice response times as a function of the Dilemma type. (**B**) Choice response times as a function of the Dilemma type and Personal Involvement. (**C**) Relation between choice response times and BIS-11 Cognitive Instability scores as a function of Dilemma type. Error bars (and shaded areas) represent standard errors of the means. TP: Time Pressure group. noTP: no Time Pressure group

The model on choice response times (R^2^ marginal = 0.250; R^2^ conditional = 0.588; Fig. 2A; Supplementary Material Table S3) showed significant main effects of *group* (*F*(1, 51) = 32.53, *p* < .001) and *dilemma type* (*F*(1, 3038) = 270.41, *p* < .001). Response times were faster in the TP than in the noTP group (noTP vs. TP: 0.463 in log scale and 4266 in msec, SE = 0.081, *t*(51) = 5.7, *p* < .001), and in footbridge- than trolley-like dilemmas (trolley vs. footbridge: 0.191 in log scale and 1169 in msec, SE = 0.012, *t*(3038) = 16.44, *p* < .001). We also found a main effect of *involvement* (*F*(1, 3038) = 4.56, *p* = .033), better specified by a significant *dilemma type* × *involvement* interaction (*F*(1, 3038) = 4.66, *p* = .031; Fig. 2B). Dilemmas with personal involvement elicited faster response times than dilemmas without personal involvement only in footbridge-like dilemmas (trolley no involvement vs. involvement: 0.00 in log scale and 26 in msec, SE = 0.016, *t*(3038) = -0.017, *p* = .987; footbridge no involvement vs. involvement: 0.05 in log scale and 210 in msec, SE = 0.016, *t*(3038) = 3.039, *p* = .002).

We also found an effect of BIS-11 Cognitive Instability score, that remained significant controlling for STAI and BDI-II scores. From the model (R^2^ marginal = 0.298; R^2^ conditional = 0.605) testing the slopes of BIS-11 Cognitive Instability trend for each level of the fixed factors, a significant interaction emerged between BIS-11 Cognitive Instability scores, *group*, and *dilemma type* (*F*(1, 3039) = 4.40, *p* = .036; Fig. 2C). The slope analysis showed that higher BIS-11 Cognitive Instability scores predicted slower response times in the noTP group to both dilemma types (trolley: β = 0.062, SE = 0.030, CI = [0.001, 0.122]; footbridge: β = 0.077, SE = 0.030, CI = [0.016, 0.138]), whereas slopes in the TP group were not statistically different from 0 (trolley: β = -0.016, SE = 0.034, CI = [-0.084, 0.051]; footbridge: β = -0.034, SE = 0.034, CI = [-0.102, 0.034]).

### Valence ratings

**Fig. 3:**
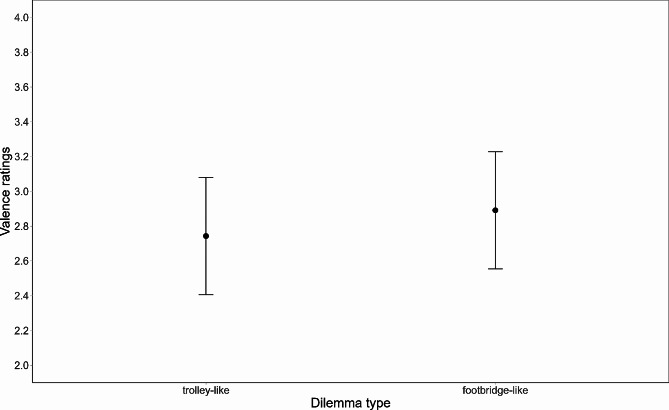
Valence ratings as a function of the Dilemma type. Error bars represent standard errors of the means

The model on valence ratings (R^2^ marginal = 0.006; R^2^ conditional = 0.485; Fig. 3; Supplementary Material Table S4) showed only a significant main effect of *dilemma type* (*F*(1, 3038) = 10.402, *p* = .001), with trolley-like dilemmas eliciting higher unpleasantness than footbridge-like dilemmas (trolley vs. footbridge: -0.148, SE = 0.046, *t*(3042) = -3.22, *p* = .001). Neither personal involvement nor questionnaire scores showed any significant effects.

### Arousal ratings

**Fig. 4:**
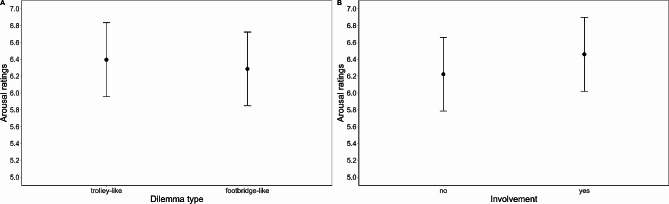
(**A**) Arousal ratings as a function of the Dilemma type. (**B**) Arousal ratings as a function of Personal Involvement. Error bars represent standard errors of the means

The model on arousal ratings (R^2^ marginal = 0.004; R^2^ conditional = 0.540; Fig. 4A; Supplementary Material Table S5) showed a main effect of *dilemma type* (*F*(1, 3038) = 4.38, *p* = .036): trolley-like dilemmas elicited higher arousal ratings than footbridge-like dilemmas (trolley vs. footbridge: 0.111, SE = 0.054, *t*(3038) = 2.09, *p* = .036). A main effect of *involvement* (*F*(1, 3038) = 20.161, *p* < .001) also emerged, with dilemmas with personal involvement eliciting higher arousal ratings than dilemmas without personal involvement (no involvement vs. involvement: -0.24, SE = 0.053, *t*(3038) = -4.49, *p* < .001). No questionnaire scores significantly modulated arousal ratings.

## Discussion and conclusions

Making timely moral decisions can be crucial in saving lives (e.g., physicians and nurses during surgeries, airline pilots during turbulent flights). However, little is known about the processes underlying moral decision-making under time pressure, and their interaction with individual differences in impulsivity or sensitivity to reward and punishment. With this study, we aimed to cover these gaps by investigating the influence of these trait dimensions on moral decision-making under time pressure.

In line with the dual-process model [1], we found that trolley-like dilemmas elicited a higher proportion of utilitarian choices and slower response times, suggesting that rational cost-benefit analysis required additional time and cognitive effort. Moreover, contrary to the dual-process framework, but consistent with prior work using the present dilemma set [31], higher unpleasantness and arousal were reported in trolley-like dilemmas. We can interpret this result as due to the higher proportion of utilitarian choices in trolley-like dilemmas. Indeed, from the qualitative analysis of descriptive statistics about valence and arousal ratings as a function of the type of choice (see Supplementary Material Table S1 and Figure S1), it seems that in both groups and dilemma types higher unpleasantness is related to a higher proportion of utilitarian choices. This suggests that sacrificing one person, even when perceived as a side effect of maximising the number of lives saved, still carries an ongoing emotional cost. Given that utilitarian choices are more numerous in trolley-like dilemmas, we can reasonably speculate that the higher unpleasantness and arousal ratings found in trolley-like dilemmas are due to the higher number of choices in which participants faced the emotional cost of utilitarian resolutions. Consistent with the dual-process model, we also found that dilemmas with personal involvement (especially the footbridge-like ones) elicited a higher proportion of utilitarian choices, faster response times, and heightened arousal. In these dilemmas, where the utilitarian option implies saving one’s own life, greater emotional engagement results in clear-cut and prompt utilitarian decisions.

However, in contrast with our hypothesis, time pressure per se did not affect moral decisions or emotional experience, as evidenced by the lack of a group effect on type of choice, valence, and arousal ratings. In particular, time pressure did not induce a heightened state of arousal, as might be expected (e.g [32]). We might speculate that emotional arousal was primarily influenced by the task of resolving dilemmas. The strong emotional engagement elicited during dilemma resolutions may have limited the impact of time pressure on subjective arousal, such that the additional stress of time constraints did not lead to a significant incremental effect. Indeed, arousal ratings were consistently high (i.e., > 6.2) across all types of dilemmas and involvement conditions in both groups.

Overall, we can reasonably rule out that these results are due to a failure in our experimental manipulation to induce time pressure. Indeed, the TP group showed faster decision times than the noTP group. It is possible that the 8-sec constraint we employed was not stringent enough to affect the type of choice (cf. 4.4-sec and 1-sec in [6, 10]). However, as in [8], our 8-sec time constraint included the reading time for the utilitarian option (∼ 6.5 s, see [3]), and the decision time was constant across dilemmas since number of words and text characters of utilitarian options was fully balanced throughout (see [4]). This meant that participants actually had only 1.5 s, on average, to make a decision. Still, it could also be conceivable that such a duration was inadequate to induce either a heightened state of arousal (as noted above) or a significant reduction in cognitive resources available for engaging controlled processes during decision-making. Indeed, previous research on moral dilemmas [33] has demonstrated that a moderate cognitive load induced by a secondary task (i.e., a concurrent digit-search task) increased response times for utilitarian judgments, while not affecting the type of judgement. Interestingly, a higher degree of cognitive load (i.e., performing an extremely difficult dot memory task) was found to be effective in reducing the number of utilitarian responses in high-conflict moral dilemmas [34]. These findings suggest that stronger experimental manipulations are needed to impact effortful cognitive processing during the resolution of moral dilemmas, either by employing a higher cognitive load or imposing stricter temporal constraints.

Nonetheless, in our view, what may explain our unexpected result of a null group effect lies mainly in the moral task employed. While previous studies [6–10, 12] used a moral acceptability question format, thus measuring moral judgments, we asked participants whether they would actually perform the proposed action. Prior research highlighted a dissociation between moral judgement and choice of action, so that the latter is more closely tied to emotional experience and personal responsibility, whereas judgement mainly relies on cognitive perspective-taking [35]. It is thus plausible that time pressure interferes more with moral judgement, which additionally requires shifting from a first- to a third-person perspective [36].

However, in our study time pressure *indirectly* influenced moral choices, as demonstrated by the interaction effects on a specific facet of attentional impulsivity. Higher scores of cognitive instability, as indexed by the BIS-11 subscale, predicted slower response times in the noTP group. Although such a result may seem counterintuitive and is in contrast with our hypothesis, previous research showed that impulsive individuals tend to be slower in choice reaction time tasks [37] and Go/NoGo tasks [38], especially when information-processing demands and response complexity are increased [39], while other studies (e.g [40]), reported an increase in time taken to resolve interference. Cognitive instability involves intrusive thoughts and rapid shifts in attention and thinking, which can lead to difficulties in maintaining a consistent approach to complex problems. Therefore, when faced with moral dilemmas, individuals with high cognitive instability might find it challenging to decide on a course of action. This could result in longer response times especially when there are no time constraints, as they might re-evaluate available choices and decision outcomes multiple times. Conversely, when the time available for decision-making was constrained (TP group), cognitive instability exerted no influence. Thus, it can be speculated that time pressure might override the (disturbing) influence of cognitive instability by promoting focus on the task at hand, minimising the impact of internal distractions, and ensuring attention is maintained on relevant information. Interestingly, these effects appear to be specific to this facet of attentional impulsivity, as other dimensions of impulsivity showed no associations with decision times. Furthermore, our analysis accounted for symptoms of depression and anxiety.

With regards to BIS-BAS, a heightened tendency to anticipate and desire immediate reward (as indexed by the BAS Reward Responsiveness subscale) predicted a higher proportion of utilitarian choices in both groups, consistent with Moore and colleagues (2011) [20]. Therefore, regardless of the time available for decision-making, individuals who are more sensitive to rewards may be more inclined towards utilitarian responses as they may prioritise the maximum overall positive outcome (i.e., saving the majority of people). This result might seem at odds with the idea that higher reward responsiveness should be related to a clear-cut prioritisation of self-interest, which, in this context, pertains to personal survival. Indeed, a number of studies on both healthy (e.g [41]), and clinical populations (e.g [42]), have highlighted that reward sensitivity plays a significant role in increasing the propensity for immoral behaviour (e.g., voluntary deception for one’s own benefit). Concurrently, research has demonstrated that individuals high in the psychopathy trait, which is associated with alterations of the neural reward system (e.g [43]), show an increased willingness to endorse utilitarian choices (e.g [35, 44]). This propensity, though, may be attributed in psychopathy to a weaker sensitivity to consequences and a reduced concern for inflicting harm [45]. In our view, in the case of sacrificial moral dilemmas, where each choice involves the death of human beings and no choice is truly “right” or definitively moral, individuals high in reward sensitivity might still find it rewarding to help others, thus pursuing social rewards. Our findings contribute to the understanding of the complex interplay between reward sensitivity and moral behaviour, highlighting the significance of a specific contextual reward condition in which the lives of other people are at stake. Interestingly, while impulsivity has been also suggested to involve a tendency to prioritise immediate over delayed rewards (e.g [46]), this trait did not influence responses or decision times specifically related to rewards, whether perceived as the saving of a greater number of lives or as personal survival. This suggests that, at least in the context of sacrificial moral dilemmas, reward-reactivity might be related yet distinct from trait impulsivity (see [47]). Contrary to Moore et al. (2011) [20], we did not find any significant effect of BIS sensitivity. This discrepancy could once again be attributed to the different processes involved in formulating a moral judgement (as in [20]) vs. in deciding to undertake an action, as in our case. The BIS, being more focused on avoiding negative outcomes, might not have been as influential in this context, where either dilemma choice had aversive implications from a first-person perspective.

Summarising, our study revealed that the impact of time pressure on moral decision-making might be more complex and multifaceted than expected, potentially interacting with a specific facet of attentional impulsivity. When dilemma resolutions are formulated as actions to be endorsed or rejected based on a first-person perspective, decision choices do not appear to be influenced by the time available for deliberation. This indicates marked stability in behavioural responses to footbridge- and trolley-like dilemmas, as well as in the respective underlying processes. However, time pressure seemed to counteract the slowing effects of individual cognitive instability, possibly by maintaining attentional focus and thus reducing the interference from cognitive-emotional conflicts. Interestingly, individual sensitivity to reward predicted overall utilitarian choices, indicating that within sacrificial moral dilemmas, the number of lives saved can be effectively reframed as a (social) reward to be pursued. As might be expected, this broad effect was not sensitive to time pressure.

Concluding, some limitations of our study are worth mentioning. First, our paradigm did not include a “question screen” (e.g [8]), that typically follows the option and to which decision times would be time-locked. This decision was based on the idea that decision-making encompasses dynamic, overlapping processes beginning as early as the reading of the option starts. However, this implied that decision times were strictly dependent on reading times. Therefore, shorter individual reading times in the TP groups might have prevented the allotted decision time from exerting sufficient pressure. Second, although our study focused on impulsivity and BIS-BAS sensitivity, and controlled for levels of anxiety and depression, we acknowledge that other temperamental or personality traits may affect the relation between time pressure and moral choices as well. We encourage further studies to better understand this complex, multifaceted phenomenon by overcoming the limitations of the present research.

### Electronic supplementary material

Below is the link to the electronic supplementary material.

Supplementary Material 1

## Data Availability

All the data and analyses cited in this manuscript have been made publicly available within the Open Science Framework (OSF) and can be accessed at the following permanent anonymous link: https://osf.io/23mwd/?view_only=ff1dafdf6c8f4e7a89bba29830d77910.

## Abbreviations

BAS: Behavioural Activation System
BDI-II: Beck Depression Inventory-II
BIS-11: Barratt Impulsiveness Scale 11
BIS: Behavioural Inhibition System
DV: Dependent variable
FDR: False discovery rate
GLMM: Generalised Linear Mixed-effects Model
LMM: Linear Mixed-effects Model
M: Mean
MAD: Median absolute deviation
noTP: No time pressure group
RT: Response time
SAM: Self-Assessment Manikin
SD: Standard deviation
SE: Standard error
STAI: State-Trait Anxiety Inventory
TP: Time pressure group
