# Supplementary material for: Pulling the lever in a hurry: the influence of impulsivity and sensitivity to reward on moral decision-making under time pressure
Source: BMC Psychol. 2024 May 14;12:270. doi: 10.1186/s40359-024-01773-y (PMC11092183; doi:10.1186/s40359-024-01773-y)
Supplement: Supplementary file 1 — Supplementary Material 1 [file 40359_2024_1773_MOESM1_ESM.pdf]

SUPPLEMENTARY MATERIAL

Pulling the lever in a hurry: the influence of impulsivity and sensitivity to reward on moral decision-making under time pressure

Group	Dilemma type	Choice type	Choice response time	Valence	Arousal
TP	trolley-like	Non-utilitarian	5897.49	2.85	6.41
		Utilitarian	5662.64	2.88	6.47
	footbridge-like	Non-utilitarian	4831.51	2.99	6.19
		Utilitarian	5325.13	3.06	6.86
noTP	trolley-like	Non-utilitarian	9857.94	2.68	6.40
		Utilitarian	10588.63	2.60	6.34
	footbridge-like	Non-utilitarian	7985.52	2.89	6.05
		Utilitarian	11907.75	2.38	6.99

Table S1. Mean choice response times (in msec), and valence and arousal ratings for each type of choice (utilitarian vs. non-utilitarian), dilemma type (trolley- vs- footbridge-like), and group (TP vs. noTP).

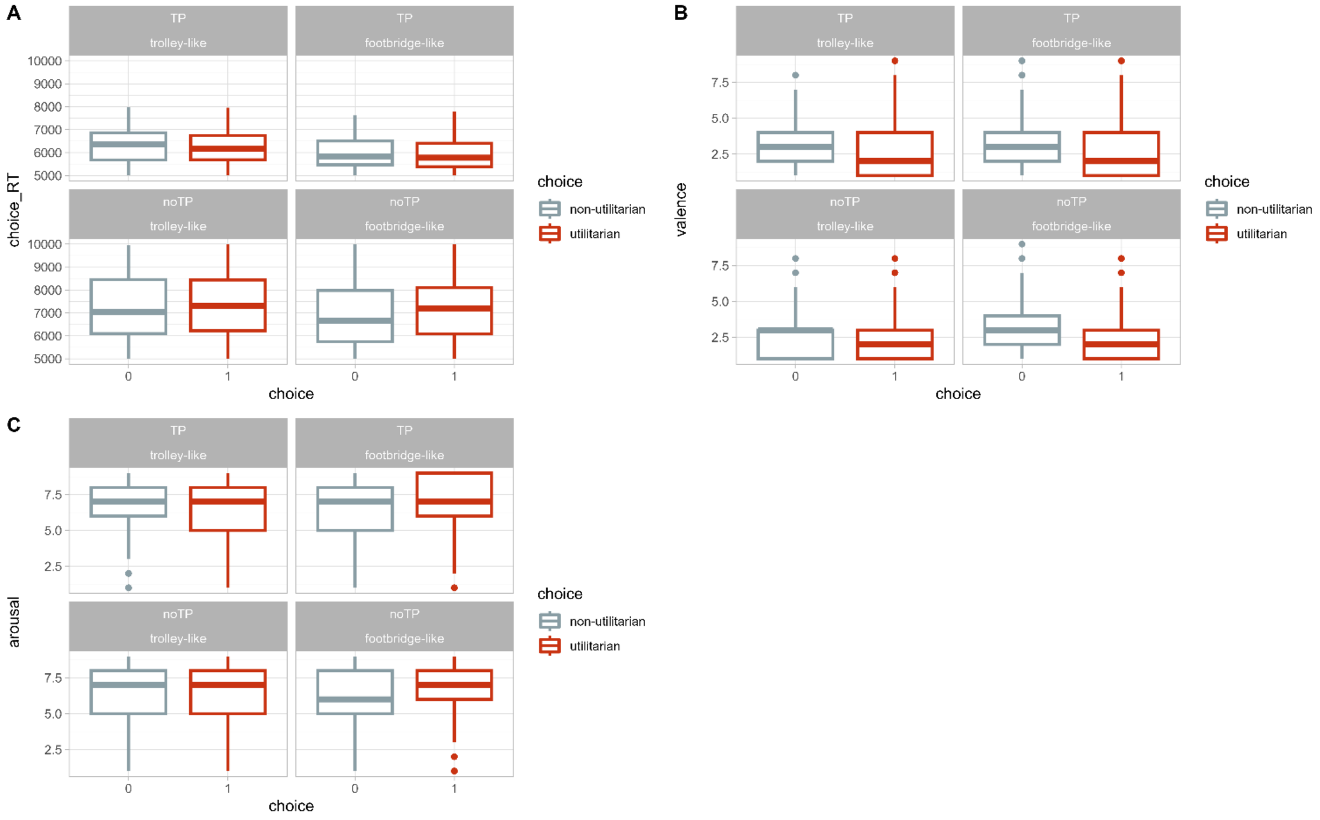

Figure S1. Boxplots of (A) choice response times (in msec), (B) valence, and (C) arousal ratings for each type of choice (0 = non-utilitarian, 1 = utilitarian), dilemma type (trolley- vs. footbridge-like) and group (TP vs. noTP).

In Table S1 and Figure S1, we report descriptive statistics and plots about choice response times, valence, and arousal ratings depending on the group (TP vs. noTP), the dilemma type (trolley- vs. footbridge-like), and the type of choice (utilitarian vs. non-utilitarian). In line with the dual-process theory of moral judgment, we should expect non-utilitarian resolutions to be faster than utilitarian ones, given that they rely more on fast, automatic emotional processes rather than on a slow, deliberate cost-benefit computation, and that this effect should be even more pronounced if we reduce the time allotted for the decision (as in the TP condition). From the qualitative observation of the descriptive statistics and plots, this hypothesis is only partially confirmed: participants showed faster choices to non-utilitarian resolutions in all dilemma types in the noTP group but only in footbridge-type dilemmas in the TP group. In trolley-like dilemmas, instead, participants in the TP group decided faster in the case of utilitarian resolutions. Thus, time pressure seems to facilitate the typical response strategy according to the dilemma type (utilitarian in trolley-like dilemmas and non-utilitarian in footbridge-like dilemmas) rather than directly modifying the type of processes (cognitive vs. emotional) involved in the choice.

Moreover, descriptive statistics suggest that decisions leading to utilitarian resolutions elicited more unpleasantness in both groups and dilemma types, and higher arousal in both groups only in footbridge-like dilemmas. These additional considerations may add further clarification to our interpretation of why trolley-like dilemmas elicited higher unpleasantness and arousal. Indeed, the qualitative commentary we offered above supports the conclusion that utilitarian resolutions (i.e., sacrificing one life), even when they are the side-effect of maximizing the number of lives saved, still carry an unpleasant emotional cost. Given that utilitarian choices are more numerous in trolley- than in footbridge-like dilemmas, we can reasonably speculate that the higher unpleasantness and arousal ratings found for trolley-like dilemmas are due to the higher number of choices in which participants faced the emotional cost of utilitarian resolutions.

Although these considerations must remain speculative since they are not supported by statistical evidence, we believe that they may be of interest to the readers of this paper.

In Tables S2-S5, we report models' summaries on choice (Table S2), choice response times (Table S3), valence (Table S4) and arousal ratings (Table S5). For each model, we report regression coefficients, the associated 95% confidence intervals (CI) and p-values (in bold when significant). For each model, significant main and interaction effects are reported in the Results section.

**Choice**			
Predictors	*Odds Ratios*	*CI*	*p*
(Intercept)	0.75	0.53 – 1.07	0.110
group	1.05	0.74 – 1.49	0.777
dilemma type	3.82	3.44 – 4.23	**<0.001**
group × dilemma type	1.00	0.91 – 1.11	0.954
involvement	0.85	0.78 – 0.93	**0.001**
group × involvement	0.96	0.88 – 1.05	0.376
dilemma type × involvement	1.16	1.06 – 1.27	**0.002**
group × dilemma type × involvement	0.94	0.86 – 1.03	0.167
**Random Effects**			
σ^2	3.29		
τ_{00}	1.53 $_{id}$		
ICC	0.32		
N	53 $_{id}$		
Observations	3097		
Marginal R^2 / Conditional R^2	0.277 / 0.506		

Table S2. Results of LMM on the proportion of utilitarian choices. For each model, we report regression coefficients (in odds ratios), and associated 95% confidence intervals (CI) and *p*-values (in bold when significant).

**Choice response time**			
Predictors	*Estimates*	*CI*	*p*
(Intercept)	8.78	8.70 – 8.86	**<0.001**
group	-0.23	-0.31 – -0.15	**<0.001**
dilemma type	0.10	0.08 – 0.11	**<0.001**
group × dilemma type	-0.01	-0.02 – 0.00	0.081
involvement	0.01	0.00 – 0.02	**0.033**
group × involvement	0.00	-0.01 – 0.01	0.805
dilemma type × involvement	-0.01	-0.02 – -0.00	**0.031**
group × dilemma type × involvement	0.00	-0.01 – 0.01	0.973
type of choice			
group × type of choice			
dilemma type × type of choice			
group × dilemma type × type of choice			
**Random Effects**			
σ^2	0.10		
τ_{00}	0.09 $_{id}$		
ICC	0.45		
N	53 $_{id}$		
Observations	3097		
Marginal R^2 / Conditional R^2	0.250 / 0.588		

Table S3. Results of LMM on choice response times. For each model, we report regression coefficients, and associated 95% confidence intervals (CI) and *p*-values (in bold when significant).

**Valence ratings**			
Predictors	*Estimates*	*CI*	*p*
(Intercept)	2.82	2.49 – 3.16	**<0.001**
group	0.11	-0.23 – 0.44	0.524
dilemma type	-0.07	-0.12 – -0.03	**0.001**
group × dilemma type	0.02	-0.03 – 0.06	0.405
involvement	0.00	-0.04 – 0.05	0.934
group × involvement	0.01	-0.04 – 0.05	0.700
dilemma type × involvement	-0.00	-0.05 – 0.04	0.931
group × dilemma type × involvement	0.01	-0.03 – 0.06	0.619
**Random Effects**			
σ^2	1.62		
τ_{00}	1.51 $_{id}$		
ICC	0.48		
N	53 $_{id}$		
Observations	3097		
Marginal R^2 / Conditional R^2	0.006 / 0.485		

Table S4. Results of LMM on valence ratings. For each model, we report regression coefficients, and associated 95% confidence intervals (CI) and *p*-values (in bold when significant).

**Arousal ratings**			
Predictors	*Estimates*	*CI*	*p*
(Intercept)	6.34	5.91 – 6.78	**<0.001**
group	0.04	-0.39 – 0.48	0.853
dilemma type	0.06	0.00 – 0.11	**0.036**
group × dilemma type	0.01	-0.05 – 0.06	0.818
involvement	-0.12	-0.17 – -0.07	**<0.001**
group × involvement	-0.03	-0.08 – 0.02	0.233
dilemma type × involvement	-0.02	-0.08 – 0.03	0.391
group × dilemma type × involvement	0.01	-0.04 – 0.06	0.781
**Random Effects**			
σ^2	2.21		
τ_{00}	2.57 $_{id}$		
ICC	0.54		
N	53 $_{id}$		
Observations	3097		
Marginal R^2 / Conditional R^2	0.004 / 0.540		

Table S5. Results of LMM on arousal ratings. For each model, we report regression coefficients, and associated 95% confidence intervals (CI) and *p*-values (in bold when significant).
